# Transcriptomic analysis of polyketide synthases in a highly ciguatoxic dinoflagellate, *Gambierdiscus polynesiensis* and low toxicity *Gambierdiscus pacificus*, from French Polynesia

**DOI:** 10.1371/journal.pone.0231400

**Published:** 2020-04-15

**Authors:** Frances M. Van Dolah, Jeanine S. Morey, Shard Milne, André Ung, Paul E. Anderson, Mireille Chinain

**Affiliations:** 1 Marine Genomics Core, Hollings Marine Laboratory, Charleston, SC, United States of America; 2 Charleston Computational Genomics Group, Department of Computer Science, College of Charleston, Charleston, SC, United States of America; 3 Laboratoire des Biotoxines Marines, Institut Louis Malardé—UMR 241 EIO, Papeete, Tahiti, French Polynesia; University of Cambridge, UNITED KINGDOM

## Abstract

Marine dinoflagellates produce a diversity of polyketide toxins that are accumulated in marine food webs and are responsible for a variety of seafood poisonings. Reef-associated dinoflagellates of the genus *Gambierdiscus* produce toxins responsible for ciguatera poisoning (CP), which causes over 50,000 cases of illness annually worldwide. The biosynthetic machinery for dinoflagellate polyketides remains poorly understood. Recent transcriptomic and genomic sequencing projects have revealed the presence of Type I modular polyketide synthases in dinoflagellates, as well as a plethora of single domain transcripts with Type I sequence homology. The current transcriptome analysis compares polyketide synthase (PKS) gene transcripts expressed in two species of *Gambierdiscus* from French Polynesia: a highly toxic ciguatoxin producer, *G*. *polynesiensis*, *versus* a non-ciguatoxic species *G*. *pacificus*, each assembled from approximately 180 million Illumina 125 nt reads using Trinity, and compares their PKS content with previously published data from other *Gambierdiscus* species and more distantly related dinoflagellates. Both modular and single-domain PKS transcripts were present. Single domain β-ketoacyl synthase (KS) transcripts were highly amplified in both species (98 in *G*. *polynesiensi*s, 99 in *G*. *pacificus*), with smaller numbers of standalone acyl transferase (AT), ketoacyl reductase (KR), dehydratase (DH), enoyl reductase (ER), and thioesterase (TE) domains. *G*. *polynesiensis* expressed both a larger number of multidomain PKSs, and larger numbers of modules per transcript, than the non-ciguatoxic *G*. *pacificus*. The largest PKS transcript in *G*. *polynesiensis* encoded a 10,516 aa, 7 module protein, predicted to synthesize part of the polyether backbone. Transcripts and gene models representing portions of this PKS are present in other species, suggesting that its function may be performed in those species by multiple interacting proteins. This study contributes to the building consensus that dinoflagellates utilize a combination of Type I modular and single domain PKS proteins, in an as yet undefined manner, to synthesize polyketides.

## Introduction

Benthic dinoflagellates of the genus *Gambierdiscus* are the primary source of ciguatoxins, neurotoxins responsible for ciguatera poisoning (CP), the most prevalent seafood toxin-associated illness in the world [1, 2]. *Gambierdiscus* spp. occur in coral reef ecosystems worldwide where they are typically found as epiphytes on macroalgae and sea grass and in benthic assemblages on coral rubble and sand. Ciguatoxins are introduced into the foodweb when herbivores graze on macroalgae that are heavily colonized by *Gambierdiscus* populations. Because ciguatoxins and their precursor gambiertoxins are lipophilic, they are accumulated, biotransformed, and biomagnified through the foodweb to top predators, including reef fish that are harvested by commercial and recreational fisheries. Ciguatoxins bind to voltage-gated sodium channels, present in brain, skeletal muscle, heart, peripheral nervous system, and sensory neurons, causing voltage independent activation and prolonged opening of the channels, which results in spontaneous and repetitive action potentials that alter sensorimotor conduction [1]. In humans, their consumption results in CP, with acute symptoms that may include diarrhea, vomiting, muscular and joint aches, numbness and tingling of the mouth and extremities, cold allodynia, irregular heartbeat, and rarely, respiratory paralysis [1,2]. Approximately 20 percent of CP cases progress to chronic ciguatera, with debilitating symptoms similar to chronic fatigue syndrome that may last from months to years [3].

Management of CP is difficult because its occurrence in reef ecosystems is patchy and associated with complex assemblages of multiple *Gambierdiscus* species that vary spatially and temporally. Eighteen species of *Gambierdiscus* are now recognized worldwide, with eight new species published since the last major review [4–9]. To date more than 50 congeners of ciguatoxin have been identified [10, 11, and references therein], and the toxin profiles in *Gambierdiscus* species can differ substantially, resulting in highly divergent toxicity between species. The toxicity of a reef area is now recognized to depend primarily on the presence of selected highly toxic species of *Gambierdiscus* that may not be the numerically dominant species [12, 13], but which contribute disproportionately to the overall toxicity of the region. In the tropical Pacific Ocean, the most toxic species known is *G*. *polynesiensis*, with a complex congener profile that includes CTX3C, a potent ciguatoxin congener found in fin fish. Most, if not all species of *Gambierdiscus* also produce maitotoxins, large water-soluble polyethers that may contribute to ciguatera-like symptoms associated with eating herbivorous fishes, but do not appear to be biomagnified in the foodweb.

Ciguatoxins and maitotoxins are members of the ladder-like polyethers, a class of polyketide that are produced primarily by dinoflagellates. Numerous labeling studies have confirmed that dinoflagellate ladder polyethers are produced by polyketide synthases (PKS) [14–17]. PKS are structurally analogous to fatty acid synthases (FAS), in which a starting substrate, acetyl CoA, is incorporated into long polyethers through a series of sequential condensations with malonyl CoA that are performed by KS domains of the PKS. In the synthesis of fatty acids, each added acetate unit undergoes ketoreduction (KR), dehydration (DH), and enoyl reduction (ER), resulting in a fully saturated carbon chain. In contrast, polyketide synthase modules may lack one or more of these catalytic domains. Thus, PKS can produce a variety of carbon chains that may include carbonyl groups (absence of KR domain), hydroxyl groups (absence of DH domain), or double bonds (absence of ER domain) [18]. The natural product potential in dinoflagellates is further enhanced by the presence of hybrid nonribosomal peptide synthases (NRPS)/PKS, that incorporate both acyl and aminoacyl building blocks into their products [19, 20].

In both FAS and PKS, the growing carbon chain is carried by an acyl carrier protein (ACP) as it is acted upon by each catalytic domain in succession. A critical player is the AT, which presents the extender units (most often malonyl coA) to the KS domain to be added to the growing chain. The full-length polyketide is released from the PKS complex by a TE. Two major groups of PKS are found. In Type I PKS all catalytic domains are found on a single polypeptide, which are used in a processive fashion for chain elongation. This structure is analogous to FASs in animals and fungi [21, 22]. Type II PKSs are multiprotein complexes where each catalytic domain is found on a separate polypeptide, analogous to type II FASs in bacteria and plants.

PKSs with sequence homology to Type I have been identified in a wide array of dinoflagellate species using PCR [23], Sanger [24, 25], and 454 [26], revealing unusual Type I transcripts bearing a single catalytic domain (eg., KS), rather than the usual multidomain structure (e.g., KS-AT-DH-ER-KR). Only recently, with the advent of deeper transcriptome sequencing afforded by RNAseq, have multidomain PKSs been identified in dinoflagellate transcriptomes, including several species of *Gambierdiscus* [27, 28] *Karenia brevis* [29], *Symbiodinium* [30, 31], and *Ostreopsis* spp. [32].

The current study conducted a comparative analysis of the transcriptomes of two *Gambierdiscus* species from French Polynesia, a highly ciguatoxic *G*. *polynesiensis* isolated from the Australes Archipelago (strain TB92), and a co-occurring low- or non-toxic species, *G*. *pacificus* (MUR4), with the goal of identifying the diversity of PKS transcripts expressed in common or unique to their very different toxin profiles. *G*. *polynesiensis* TB92 produces Pacific ciguatoxins of Type 1 (P-CTX4A, P-CTX4B) and Type 2 (P-CTX3C, 49-epi-P-CTX3C, M-seco-P-CTX3C, M-seco-P-CTX 4A) ladder structures [33, 34]. This species also produces 44-methyl gambierone (44MG, formerly known as MTX3) [35], a ubiquitous polyether compound found in the genera *Gambierdiscus* and *Fukuyoa* [13, 36, 37]. In contrast, *G*. *pacificus* does not appear to produce ciguatoxins. To broaden the comparison, we mined publicly available sequences from two maitotoxin-producing species *G*. *australes* and *G*. *belizeanus* [27], as well as two additional high toxicity CTX and MTX-producers, *G*. *excentricus* from Tenerife Island, Spain, and a second strain of *G*. *polynesiensis* (CAWD212), isolated from the Cook Islands [28].

We found that both species expressed highly amplified numbers of single domain PKS transcripts, many of which appear to have homologs in the other species. However, the highly ciguatoxic *G*. *polynesiensis* expressed a larger number of multidomain PKSs, with larger numbers of modules, than the non-ciguatoxic *G*. *pacificus*. The largest modular PKS in *G*. *polynesiensis* from the Australes Archipeligo contained 7 modules, consistent with the findings of Kohli et al. [28], who identified a similar 7-module multidomain PKS in *G*. *polynesiensis* from the Cook Islands.

## Methods

### Strains and culture conditions

*G*. *polynesiensis* strain TB92 was isolated from a 1992 bloom in Tubuai Island, Australes Archipelago, French Polynesia [33]. *G*. *pacificus* strain MUR4 was isolated in 2005 from Moruroa Atoll, Tuamotu Archipelago, French Polynesia [33]. Both non-axenic isolates were grown under conditions previously used to characterize toxicity and toxin profiles [33]. Cultures were maintained in 1 L fernbach flasks containing f10K enriched natural seawater medium at 27°C with a light:dark photoperiod of 12h:12h (light at 50 μmol photons m^-2^ s^-1^). Cells were harvested in late exponential phase by filtration through a sieve of 40 μm porosity, then centrifuged at 600 x *g* for 5 min at 4°C. The supernatant was discarded and cell pellets were immediately frozen in liquid nitrogen and stored in -80°C until further analysis.

### RNA extraction

Cells were disrupted, on ice, in the presence of 1 ml chilled TRI Reagent and 0.5 mm zirconium beads using a Mini-BeadBeater-1 Homogenizer (BioSpec,OK, USA). The resulting homogenates were removed from the beads by centrifugation. Total RNA was then extracted using the TRI Reagent manufacturer’s protocol (Molecular Research Center, Inc, Cincinnati, OH). Following isopropanol and high-salt precipitation, RNA was re-suspended in RNase-free water containing RNasin ribonuclease inhibitor (Promega, WI, USA). The RNA was then further purified using the RNeasy MinElute cleanup kit (Qiagen) with on-column DNase digestion according to the manufacturer’s recommendations. The integrity and quantity of the purified RNA were assessed using an Agilent 2100 Bioanalyzer (Agilent Technologies, CA, USA) and NanoDrop spectrophotometer (ThermoScientific, DE, USA), respectively.

### RNAseq libraries and sequencing

RNA sequencing libraries were generated using the NEBNext Ultra Directional RNA Library Prep Kit (Illumina) from total RNA. Sequencing was performed on an Illumina Hiseq 2500 sequencer, at a depth of approximately 178 million, 125 nt, single end reads per library, by NC State University’s Genomics Services Laboratory.

### Transcriptome assembly and analysis

Sequence processing and analysis were carried out in CyVerse’s Discovery Environment using the High-Performance Computing applications [38]. The Illumina BCL output files were converted to FASTQ-sanger file format and sequence quality trimming was performed using Trimmomatic [39], with a minimum phred quality score >20 over the length of the reads. The trimmed reads were then quality checked using the FASTQC tool. The processed and trimmed reads were used to construct *de novo* transcriptomes using the Trinity assembler v2.0.6 [40] on CyVerse’s Atmosphere cloud computing platform, using a minimum overlap value of 25 and a minimum contig length of 400 nucleotides (nt). Raw sequence reads and assembled transcriptomes are available from NCBI Bioproject numbers PRJNA561766 (*G*. *polynesiensis*) and PRNJA561774 (*G*. *pacificus*).

The transcriptomes were annotated using BLAST+ for blastx searches (E-value ≤ 1e-4), followed by conserved domain mapping and gene ontology assignment using Blast2GO v.4.1.9 [41]. The Core Eukaryotic Genes Mapping Approach (CEGMA) [42] and Benchmarking Universal Single Copy Orthologs (BUSCO V3.0.2) [43] were used to analyze the comprehensiveness of the gene catalogues.

HMMER [44] was used for the identification of contigs containing conserved PKS domains (KS, KR, ACP, AT, DH, ER, TE) using an in-house HMM database and an E-value cutoff of ≤10e-10. Functional prediction of sequences was further analyzed by Pfam [45] and conserved domain searches [46] were used for identification of conserved amino acid residues and functional prediction of PKS and NRPS/PKS transcripts. Phylogenetic analysis steps were performed in Geneious software [47]. Sequences were aligned using ClustalW [48]. Alignments were trimmed manually to ensure they spanned the conserved domains. Maximum likelihood phylogenetic analysis was carried out using PhyML [49] using the LG model of rate heterogeneity with 100 bootstraps. Phylogenetic trees were visualised using MEGA:Version 7 [50].

## Results and discussion

The transcriptome assembled from 178,416,876 *G*. *polynesiensis* (TB92) reads resulted in 66,611 contigs, with an N50 of 1544 nt and average sequence length of 1363 nt. The longest contig in this assembly was 31,688 nt, with 61 scaffolds >10K nt in length. The transcriptome of *G*. *polynesiensis* (MUR4) assembled from a similar number of reads (179,963,422 reads) resulted in 59,620 contigs. The N50 of *G*. *pacificus* was 1554 nt and the average sequence length was 1277 nt. In contrast to the *G*. *polynesiensis* transcriptome, the longest scaffold in the *G*. *pacificus* assembly was 8910 nt, and there were no scaffolds >10K nt in length.

The GC contents were 61.6% and 61.2% for *G*. *polynesiensis* and *G*. *pacificus*, respectively. This is similar to other *Gambierdiscus* species [28, 51] and most peridinin dinoflagellates (~60%) [32, 52], although some peridinin containing species (e.g., *Symbiodinium* 50.5%–56.4% [53]) and fucoxanthin containing dinoflagellates (e.g., *Karenia brevis* 52.4%, [29]) have lower GC content.

BLASTx searches found 51.8% of *G*. *polynesiensis* contigs and 51.1% of *G*. *pacificus* contigs with significant similarity to sequences in the Genbank non-redundant database (E value < 10^−4^). To assess the completeness of the transcriptome assemblies, we found 84.7% and 84.3% complete copies of 248 ultra-conserved core eukaryotic genes using CEGMA in *G*. *polynesiensis* and *G*. *pacificus*, respectively. BUSCO analysis revealed 81.2% (*G*. *polynesiensis*) and 82.3% (*G*. *pacificus*) of 303 highly conserved single-copy orthologs in the assemblies.

### Polyketide synthases

Because of the high sequence conservation within active sites, PKSs are most often identified by the presence of KS domains. HMMER and conserved domain searches found 107 KS domain containing contigs in the *G*. *polynesiensis* transcriptome. Of these 98 possessed a single KS domain while 9 sequences contained multiple KS domains in 1 to 7 modules. *G*. *pacificus* possessed a similar number of KS domains (103) but in contrast to *G*. *polynesiensis*, only four were found in multidomain sequences and these contained only single or partial modules (a complete list of all KS contigs is presented in S1 and S2 Tables). The number of KS transcripts and multidomain PKSs is presented in Table 1, in comparison with those found in previously reported transcriptomes from two low-toxicity, MTX producing species *G*. *belizeanus*, and *G*. *australes*, and two high toxicity CTX and MTX producing species, *G*. *exentricus* and a isolate of *G*. *polynesiensis* from the Cook Islands. Among these, the two *G*. *polynesiensis* isolates are the only transcriptomes that encoded multidomain PKSs greater than one module in length. It is of note that the previously published *G*. *polynesiensis* transcriptome [28] was based on approximately 10-fold deeper sequencing depth, compared with the other assemblies, allowing the speculation that the absence of longer multidomain sequences in other species could be due to insufficient sampling. However, the current *G*. *polynesiensis* transcriptome sequencing depth is more similar to those of the other species, yet a similar 7-module PKS was assembled. This suggests that the 7-module PKS is unique to *G*. *polynesiensis* and present in isolates from distant regions of the south Pacific, the Australes Archipelago of French Polynesia (strain TB92, this publication) and the Cook Islands (strain CAWD212) [50].

**Table 1:** Comparison of transcriptome assemblies and KS domains present in *Gambierdiscus* spp.

*Gambierdiscus* species	*polynesiensis*	*pacificus*	*polynesiensis*	*excentricus*	*australis*	*belizeanus*
Isolate	TB92	MUR4	CAWD212	VGO790	CAWD149	CCMP401
Data Source	this study	this study	Ref. [26]	Ref. [26]	Ref. [25]	Ref. [25]
Location	Tubuai Is., French Polynesia	Moruroa, French Polynesia	Rarotonga, Cook Islands	Tenerife Is., Spain	Rarotonga, Cook Islands	Barthelemy Is., Caribbean
Sequencing Instrument	HiSeq 2500	HiSeq 2500	HiSeq2000	HiSeq2000	HiSeq2000	HiSeq2000
Sequencing format	125 nt SE	125 nt SE	100 nt PE	100 nt PE	100 nt PE	100 nt PE
# input sequences	1.79E+08	1.78E+08	1.06E+09	1.35E+08	7.93E+07	6.16E+07
# contigs	66,611	59,620	115,780	77,393	83,353	84,870
Total # KS	107	103	143	106	102	114
# KS single domain contigs	98 (91.6%)	99 (97.1%)	130 (90.9%)	104 (98.1%)	95 (93.1%)	110 (96.5%)
# KS in PKS multi domain contigs	9 (8.4%)	4 (3.9%)	13 (9.1%)	2 (1.9%)	7 (6.9%)	4 (3.5%)
# KS domains per contig	1–7	1	1–7	1	1	1
Toxicity ^1^(MBA)	high	low	high	high	low	low
Toxin Profile: CTX	^2^CTX3C, CTX3B, CTX4A, CTX4B, M-seco-CTX3C, 2-hydroxy-CTX3C	^3^undetected	^4^CTX3C, CTX3B, CTX4A, CTX4B	^5^L(or M)-seco-CTX4A or 4B, L (or M)-seco-CTX3C or 3B, tetraseco-P-CTX3C or 3B	^6^^,^^7^undetected	^7^undetected
MTX and ^10^44MG	^8^44MG	^3^44MG	^4^44MG	^9^44MG, MTX4	^7^MTX1, 44MG	^7^44MG

^1^ mouse bioassay

^2^Ref. [33]

^3^in *G*. *pacificus* isolates: (Ref. [35, 36, 54])

^4^Ref. [54]

^5^tentative identification (Ref. [55])

^6^Ref. [35, 36, 54]

^7^Ref. [27]

^8^Ref. [56]

^9^Ref. [57]

^10^44-methylgambierone, previously known as MTX3 (Ref. [37])

**Fig 1:**
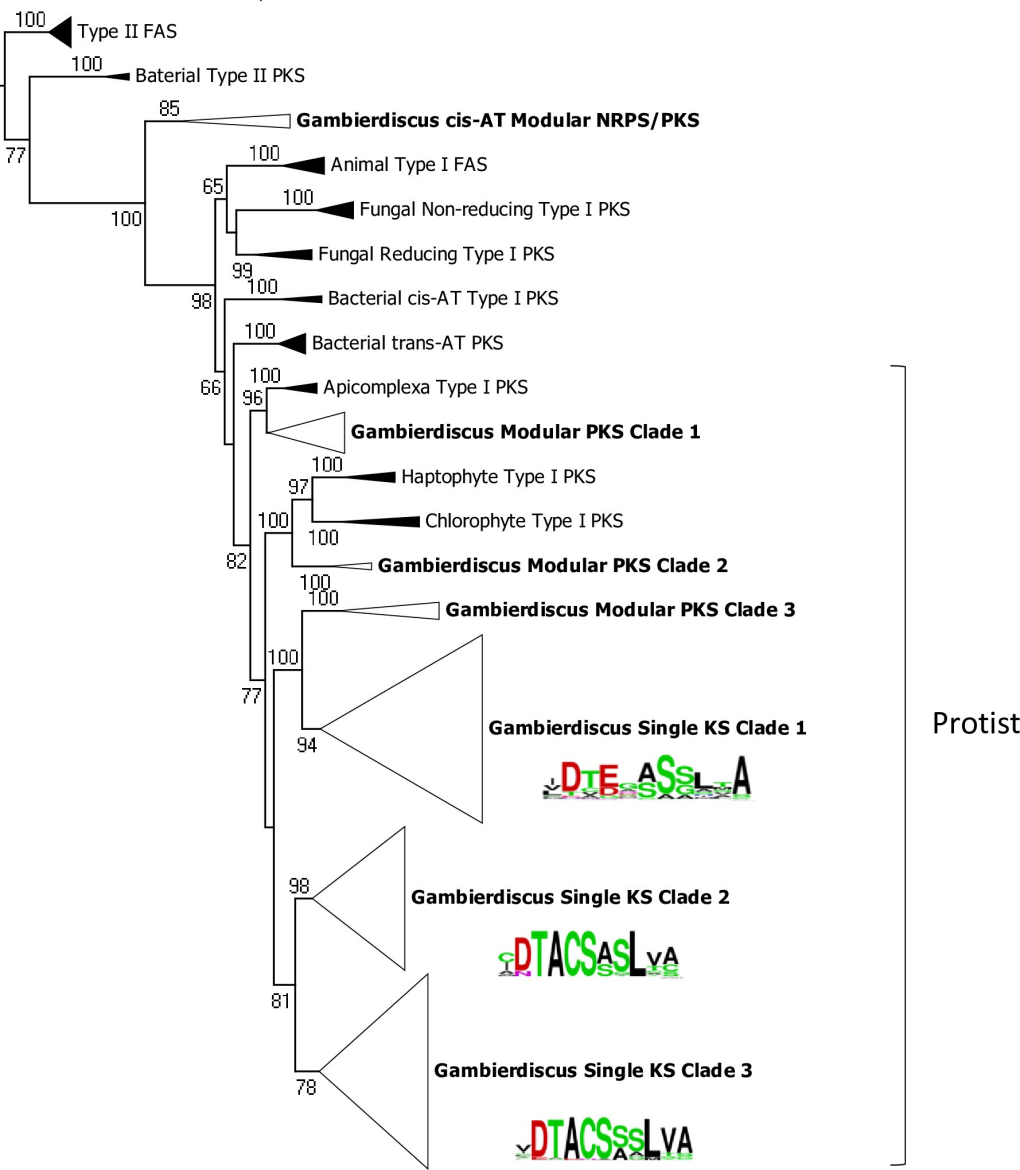
Phylogenetic analysis of *G*. *polynesiensis* and *G*. *pacificus* KS domains. The alignment consisted of 227 KS domains from *G*. *polynesiensis* TB92 and *G*. *pacificus* MUR4 and from prokaryotic and eukaryotic type I and type II PKS and FAS. Analysis was carried out by PhyML using the LG model of rate heterogeneity and 100 bootstraps. Only bootstrap values >50% are displayed.

To better define the relationships among the identified *Gambierdiscus* KS domains and those from well-studied PKS in other phyla, a maximum likelihood phylogenetic tree was constructed (Fig 1). The majority of *Gambierdiscus* KS domains fell within a clade that included Type I modular PKSs from other protists, including Apicomplexa, haptophytes, and chlorophytes, with several subclades of *Gambierdiscus* modular PKS and all standalone KS domains. *Gambierdiscus* KS domains from hybrid NRPS/PKS sequences fell in a clade outside of the protist clade and distant to other Type I PKS.

### Single domain KSs

Of the 98 *G*. *polynesiensis* transcripts containing standalone KS domains, 68 also encoded the conserved 5’ dinoflagellate-specific domain (conserved protein domain family cl22841) unique to single-domain KSs. The *G*. *pacificus* assembly contained 80 single domain sequences that included the dinoflagellate-specific domain and 19 full or partial KS sequences lacking the 5’ dinoflagellate-specific domain. The phylogenetic placement of the standalone KSs lacking the 5’ dinoflagellate-specific domain confirms that they are standalone KS domains, and not unassembled pieces of multidomain PKSs.

Phylogenetic analysis places the standalone KS domains in three clades (Fig 1), consistent with previously published analyses of dinoflagellate KSs [27, 28, 29, 32, 50]. The three clades differ in their conserved active sites, as summarized by sequence logos (Fig 1). Clade 1 active sites lack the conserved cysteine required for anchoring the growing polyketide chain in advance of decarboxylative condensation [58]; therefore, the function of these sequences remains uncertain. Clades 2 and 3 contain the conserved cysteine but vary at neighboring residues. Within each clade, almost all single domain KS sequences were found in pairs, where the closest match was a homolog found in the other species (i.e., *G*. *polynesiensis* and *G*. *pacificus*). This suggests that these unique single-domain KSs were present before the divergence of the two *Gambierdiscus* species (expandedsingle-domain clades presented in S1 Fig).

#### Clues to intracellular location of single domain KSs

We analyzed the N-terminal ends of all full length single-domain KS sequences for the presence of targeting sequences using signalP and targetP algorithms. There was no evidence for signal peptides, necessary in dinoflagellates for ER localization, secreted proteins, and plastid targeted proteins [59], or chloroplast transit sequences on any of the KS proteins. This is in contrast to fatty acid synthases in these species (fabF, TB92contig17677 Genebank Accession No. MT165605; MURcontig24072 Genebank Accession No. MT165606), which clearly possessed signal peptides, as previously reported in dinoflagellates [52].

Interestingly, a new subcellular localization tool DeepLoc [59] assigned the majority of full-length single-domain KS sequences (81% of *G*. *polynesiensis*, 98% of *G*. *pacificus*), to the peroxisome (S3 and S4 Tables). A recent transcriptome study of *Ostreopsis* spp. similarly placed a subset of KS domains in peroxisomes using DeepLoc [32]. Peroxisome targeting signals (PTS) are recognized by peroxin (Pex) proteins for import to the peroxisome. The most common is PTS1, a tripeptide consensus sequence (S/A/C)-(K/R/H)-(L/M) found at the C-terminal end of peroxisomal proteins that are imported by Pex5. A less common motif, PTS2 [(R/K)-(L/V/I)-X_5_-(H/Q)-(L/A)], is found at the N-terminal region of proteins recognized by the importer Pex7. Both manual inspection and analysis using the PTS Predictor algorithm (http://www.peroxisomedb.org) indicated the absence of these canonical PTS sequences in the N- and C-terminal ends of all single-domain KS proteins.

The DeepLoc algorithm does not look explicitly for these motifs, but rather conducts neural network analysis using a training set of 13,858 proteins with experimentally confirmed intracellular localization from the Uniprot database, including 154 peroxisome matrix and membrane proteins [60]. To better evaluate the validity the Deeploc algorithm’s assignment of KSs to the peroxisome, we searched for known peroxisomal proteins in the transcriptomes of *G*. *polynesiensis* and *G*. *pacificus* in order to characterize their PTS motifs. An inventory of peroxisomal metabolic pathways present in dinoflagellates (*Prorocentrum minimum*) and other alveolates has been recently published [61]. These include the beta oxidation of fatty acids, catabolism of purines, detoxification of reactive oxygen species, photorespiration, and the glyoxylate cycle, which uses acetyl-CoA from the breakdown of fatty acids as a carbon source for the synthesis of succinate. To identify peroxisomal proteins in our *Gambierdiscus* transcriptomes we searched for genes in this inventory by text search of Blast2Go annotations and by blasting *Prorocentrum minimum* sequences present in the inventory against our *Gambierdiscus* databases. Nearly all of the predicted peroxisomal proteins possessed PTS1 peroxisome targeting signals on their C-terminal ends (Table 2). Homologs of 3-ketoacyl thiolase in each species possessed identifiable PTS2 signals in their N-terminal ends. Consistent with these findings, both Pex5 and Pex7 homologs were identified in both transcriptomes (Pex5: TB92comp21338, MUR4comp9500; Pex7: TB92comp14151, MUR4comp7464), indicating that dinoflagellates use the conserved import mechanisms present in other eukaryotes.

**Table 2:** Conserved PTS1 C-terminal and PTS2 N-terminal containing peroxisomal proteins in *P*. *minimum* [59] and homologs found in *G*. *polynesiensis* and *G*. *pacificus*. C-terminal peroxisome targeting signal PTS1, (S/A/C)-(K/R/H)-(L/M), and N-terminal PST2 targeting signal (R/K)-(L/V/I)-X5-(H/Q)-(L/A) were found in candidate peroxisomal proteins in *Gambierdiscus*. Mito—mitochondrial; Cyto—cytoplasm. Genbank accession numbers for *Gambierdiscus* spp. peroxisome sequences are presented in S5 Table.

ID	*P*. *minimum*	Deeploc Localization	C-terminus	*G*. *polynesiensis*	Deeploc Localization	C-terminus	*G*. *pacificus*	Deeploc Localization	C-terminus
**PTS1**	*** ***			*** ***			*** ***		** **
2,4 dienoyl reductase 2	AND95769.1	Peroxisome	**SRL**	TB92 contig7114	Peroxisome	**SKL**	MUR4 contig20479	Peroxisome	**SKL**
peroxisomal 2-hydroxy acid oxidase	not found			TB92 contig49363	Peroxisome	**S**S**L**	MUR4 contig22291	Peroxisome	SN**L**
acyl coA oxidase	AND95764.1	Peroxisome	**SRL**	TB92 contig23142	Peroxisome	**SKL**	MUR4 contig28468	Peroxisome	**SKL**
dienoyl coA isomerase	AND95770.1	Peroxisome	**SKL**	TB92contig39853	Peroxisome	**SKL**	MUR4contig16107	Peroxisome	**SKL**
peroxisome multifunctional protein	AND95765.1	Peroxisome	**SKL**	TB92 contig2191	Peroxisome	**SRL**	MUR4contig28429	Peroxisome	**SKL**
peroxisomal bifunctional protein	AND95767.1	Peroxisome	**SKL**	TB92contig47060	Peroxisome	**SKL**	MUR4 contig18811	Peroxisome	**SRL**
long chain fa transporter	AND95773.1	Mito	**AKL**	TB92contig2441	Mito	**SRL**	MUR4contig10003	Mito	**SRL**
GST kappa 1	AND95780.1	Peroxisome	**AKL**	TB92 contig10582	Peroxisome	**AKL**	MUR4 contig4454	Cyto/Mito	**AKL**
**PTS2**			**N-terminal**			**N-terminal**			**N-terminal**
3 ketoacyl thiolase	AND95768.1	Peroxisome	**RL**NRLVG**QI**	TB92 contig3993	Peroxisome	**RL**QRIAK**HI**	MUR4 contig5403	Mito	**RL**QRIAQ**HV**

Given the absence of PTSs on single-domain KS sequences, and the demonstrated presence of these conserved peroxisome import pathways in *Gambierdiscus*, the basis of the Deeploc neural network assignment of the KS sequences to the peroxisome is unclear. No significant sequence homology was found between *Gambierdiscus* single domain KS sequences and proteins in the Deeploc training set of peroxisome proteins (http://www.cbs.dtu.dk/services/DeepLoc/data.php) when analyzed by Blast. A careful look at Deeploc’s performance for assignment of proteins to peroxisomes [60] indicates that only 1% of the training set is known to be peroxisomal and its sensitivity is extremely low, with the discrimination between peroxisome/cytosol often mis-classified. Thus, based on the absence of N-terminal targeting sequences and N- or C-terminal PTS motifs, all single domain PKS contigs would appear to be cytosolic.

### Modular PKSs

**Fig 2:**
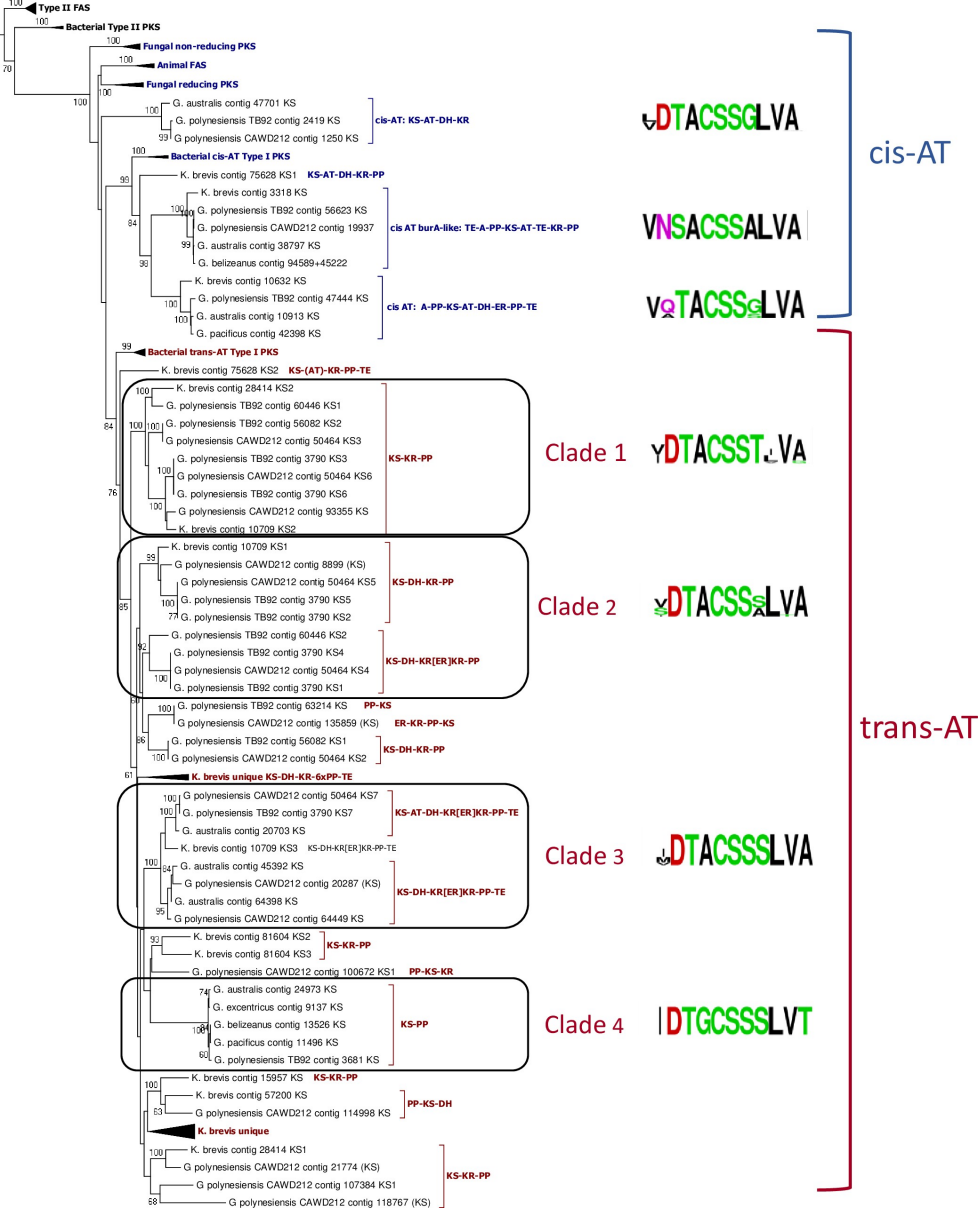
Phylogenetic analysis of KS domains extracted from modular PKS and NRPS/PKS. The alignment consisted of 116 sequences, including all modular KS from this study and previously published *Gambierdiscus* spp., *K*. *brevis*, and *cis*- and *trans*-AT prokaryotic and eukaryotic type I PKS and FAS. Type II PKS and FAS served as outgroups. Analysis carried out by PhyML using the LG model of rate heterogeneity and 100 bootstraps. Only bootstrap values >50% are displayed. Sequence logos of the active site are shown for each major clade.

The modular PKS contigs identified in *G*. *polynesiensis* and *G*. *pacificus* were predominantly of trans-AT architecture (Table 3), as previously observed in dinoflagellates [26, 27, 29] as well as other eukaryotic microalgae [62]. KS domains from *cis-*AT architectures were mainly associated with hybrid NRPS/PKS. In order to better identify modular PKS unique to or found in common among *Gambierdiscus* species, a second phylogenetic tree (Fig 2) was constructed which included the KS domains extracted from multidomain PKS sequences identified in this study as well as from all publicly available multidomain PKSs previously reported in other *Gambierdiscus* spp. [27, 28] and the phylogenetically distant dinoflagellate, *Karenia brevis* [29]. Within both the *cis-* and *trans-*AT dinoflagellate clades, KS domains tended to cluster according to the domain architecture of the module they were extracted from.

**Table 3:** Multidomain PKS in *G*. *polynesiensis* and *G*. *pacificus*. Genbank accession numbers are listed in S1 and S2 Tables.

Contig #	Length (nt)	Domain Architecture
***G*. *polynesiensis* (TB92)**		
TB92 contig3790	31688	KS-DH-KR[ER]KR-PP-PP-KS-DH-KR-PP-KS-KR-PP-KS-DH-KR[ER]KR-PP-PP-KS-DH-KR-PP-KS-KR-PP-KS-AT-DH-KR[ER]KR-PP-TE
TB92contig56082	9139	ER-KR-PP-KS-DH-KR-PP-KS-KR-PP
TB92contig60446	7530	KR-PP-KS-KR-PP-KS-DH
TB92contig47444	8197	A-PP-KS-KR-DH-ER-PP-TE
TB92contig3681	2547	KS-PP
TB92contig2419	6640	KS-AT-DH-KR
TB92contig56623	5192	KS-AT-TE-KR-PP
TB92contig60709	1987	KS-DH-KR
TB92contig63214	1617	PP-KS
TB92contig61929	2138	ER-KR
TB92contig48452	3012	ER-KR-PP-TE
TB92contig54364	5766	A-KR-PP-Leu rpt
***G*. *pacificus* (MUR4)**		
MUR4contig42398	7014	A-PP-KS-KR-DH-ER-PP-TE
MUR4contig9557	5781	AT-DH-KR[ER]KR-PP-TE
MUR4contig11496	2380	KS-PP
MUR4contig52901	1063	PP-KS
MUR4contig48072	3082	KS-AT-TE
MUR4contig34441	5355	A-KR-PP-leu rpt^1^
MUR4contig39576	1365	KR-PP
MUR4contig54128	1053	ER-KR

^1^leu rpt–leucine repeat

Two of three dinoflagellate sub-clades within the *cis-*AT were NRPS/PKS sequences. The first, with the domain structure TE-A-PP-**KS**-AT-TE-KR-PP, had similarity to the bacterial *Burkholderia* burA, previously described in a wide variety of dinoflagellates [32, 62]. Contigs containing burA-like full or partial domain arrangements were present in all *Gambierdiscus* transcriptomes. The conserved residues in the active site were identical in all members of this clade VNSA**C**SSALVA …**H**CGTG …NIA**H**, including a sequence from *Karenia brevis*. The bacterial burA adenylation domain provides three carbons from methionine to condense with malonyl CoA and has been predicted to generate propionate in a pathway common to many or all dinoflagellates [63].

The second *cis-*AT clade that consisted of NRPS/PKS sequences possessed the domain structure A-PP-**KS**-AT-DH-ER-PP-TE. Sequences in this clade were found in *G*. *polynesiensis* TB92, *G*. *pacificus*, and *G*. *australis*, as well as *K*. *brevis* and had active sites V(A/Q)TA**C**SSSLVA …**H**GGTG …N(L/V)G**H**, where the *K*. *brevis* sequence differed from the *Gambierdiscus* sequences at the amino acids listed in parentheses. When comparing the full-length sequences within this clade, *G*. *pacificus* Contig42398 had 80% identity and 90% similarity to *G*. *polynesiensis* TB92contig47444, but lacked the N-terminal adenylation domain that identified it as an NRPS/PKS. This sequence is also 80% identical (90% similarity) to *G*. *australes* sequence 10913 [25] which, after correcting a frameshift in the published sequence, had the architecture A-PP-**KS**-KR-DH-ER-PP-TE. *G*. *polynesiensis* TB92 contig47444 was nearly identical (99.5%) with *G*. *polynesiensis* CAWD212contig38791. The *K*. *brevis* contig (Kbcontig10632) with identical domain structure had 49% identity and 61% similarity with *G*. *polynesiensis* TB92contig47444. Sequences with the same architecture were also recently reported from *Ostreopsis* spp. [32] but were absent from *Symbiodinium* isolates reported in [31]. However, *S*. *microadriaticum* PKS N (Genbank Acession No. OLQ14315.1), has partial similarity in domain structure (A-PP-KS-KR), and is a top blast hit to the *Gambierdiscus* sequences in this clade.

The only other NRPK/PKS sequences found in the *Gambierdiscus* transcriptomes (A-KR-PP-leu rpt; Table 3) lack a KS domain and were therefore not included in the KS-based phylgeny. This sequence was found in both *Gambierdiscus* species. A similar sequence found in *S*. *microadriadicum* (Genbank Acession No. OLQ09666.1) is listed as a HSC70 interacting protein because of an upstream heat shock binding motif, absent from the *Gambierdiscus* sequences. The c-terminal leucine repeats are involved in protein-protein interactions.

*Trans-*AT dinoflagellate KS domains fell in a clade with the bacterial *trans-*AT KSs. Within the *trans-*AT clade, four main domain architectures were present. With the addition of sequences from other *Gambierdiscus* species, the Modular Clade 1 from Fig 1 resolved into two clades, Clade 1 and Clade 2. Clade 1 contained KS-KR-PP modules with representatives from *G*. *polynesiensis* and *K*. *brevis*, but no representatives from other *Gambierdiscus* species. Clade 2 included KS from modules that included dehydratases, KS-DH-KR-PP or KS-DH-KR[ER]KR-PP. In the latter, the ER is embedded between the two lobes of the KR domain as previously observed in *K*. *brevis* [29] and *Ostreopsis* spp. [32]. Clade 3 is made up of KSs from TE containing modules KS-DH-KR[ER]KR-PP-TE. Within this clade, one branch includes *cis-*AT modules KS-AT-DH-KR[ER]KR-PP-TE, the only *cis-*AT KS domains to occur within the larger *trans-*AT clade. Cis-AT sequences of this architecture similarly grouped with trans-AT KSs in Fig 1 (Modular Clade 2). The active site in Clade 3 cis-AT KSs (**I**DTACSSSLVA) differs from trans-AT KSs only in in one position (**V/M**)DTACSSSLVA. These differ from cis-AT PKSs sequences found in the cis-AT clade, which have an active site (**L/V**)DTACSS**G**LVA. The fourth subclade included sequences of the structure KS-PP, which were found in all *Gambierdiscus* species. Sequences with this architecture were found in clade 3 in Fig 1.

**Fig 3:**
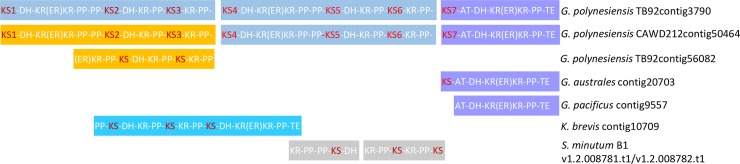
Modular PKSs sharing domain architecture and sequence homology with the 7-module PKS found in *G*. *polynesiensis*. The starting KS of each module is in red.

The phylogeny helped to elucidate the structure of the 7-module PKS found in both in *G*. *polynesiensis* isolates and identified related sequences found in other species. In both *G*. *polynesiensis* isolates, modules 1–3 were identical in organization to modules 4–6, with module 7 being unique in that it included a *cis-*AT and was followed by a TE. Modules 1, 4, and 7 included an ER domain embedded between two lobes of the KR domain, whereas modules 2 and 5 had KS-DH-KR-PP while 3 and 6 had KS-KR-PP structure. When full contigs were compared, the amino acid sequences were nearly identical between the two *G*. *polynesiensis* isolates from modules 4–7 (99.6% id), but there was significant variation (44.9% id) between isolates in modules 1–3. In *G*. *polynesiensis* TB92, modules 1–3 are nearly identical to its own modules 4–6, suggesting an origin in gene duplication (Fig 3). In contrast, modules 1–3 in *G*. *polynesiensis* CAWD212 are more closely related based on phylogeny and sequence similarity (99% id) to *G*. *polynesiensis* TB92contig56082 (Fig 3).

Sequences of the same color share >85% amino acid identity. Lighter blue shade indicates lower amino acid identity (52%) but identical domain architecture. Grey boxes indicate identical domain architecture but no data is available on amino acid identity.

Since the 7-module PKS is predicted to produce part of the backbone of polyether ladders [28], it seems to perform a function needed by all dinoflagellate ladder polyether producers. Its absence from the other dinoflagellate species may indicate that this gene function is performed by multiple smaller interacting PKS proteins, or simply that the assemblies are incomplete. The phylogeny revealed a sequence in *G*. *australes* (contig 20703, Clade 3) with 85% identity to module 7 and a similar contig was present in *G*. *pacificus* (contig9557) that lacked a KS domain (Fig 3). No sequences with similarity to other modules of the 7-domain PKS were present in the *G*. *pacificus* assembly or in the other *Gambierdiscus* species previously studied. However, the phylogeny revealed a sequence in *K*. *brevis* (Kbcontig10709) that resembled modules 2-3-4 or 5-6-7 of the *G*. *polynesiensis* 7-module PKS in both architecture and phylogenetic affinity, with a structure of PP-**KS1**-DH-KR-PP-**KS2**-KR-PP-**KS3**-DH-KR(ER)KR-TE (Fig 3). *K*. *brevis* falls in Clade 2 (Fig 2) with KS2 and KS5 of the *G*. *poynesiensis* 7-domain sequences, *K*. *brevis* KS2 falls in Clade 1 with KS3 and KS6. *K*. *brevis* KS3 falls in Clade 3 with KS7 of the *G*. *polynesiensis* sequences, and like domain 7, is followed by a TE domain. However, the *K*. *brevis* module 3 lacks an AT present in the *G*. *polynesiensis* module 7, so more closely resembles module 4. The *K*. *brevis* sequence has 65% amino acid similarity (52% identity) with *G*. *polynesiensis*TB92 modules 2-3-4 and 69% similarity (56% identity) with modules 5-6-7 if the AT gap is removed. Genomic sequencing of *Symbiodinium minutum* B1 [30], identified two adjacent gene models on the same genomic scaffold that encoded transcripts with domain structures identical to parts of modules 1–4 or 4–7 (Fig 3).

The phylogeny included a clade unique to *K*. *brevis* with PKS contigs containing highly amplified PP domains, present in six consecutive repeats [29]. In *Gambierdiscus* spp., tandemly repeated PP binding domains were observed only in the 7-domain PKS of *G*. *polynesiensis*, following domain 1 and 4, and only as duplicate repeats.

### Survey of other PKS domains

HMMR and conserved domain searches were used to identify contigs containing additional PKS domains, including KR, AT, DH, ER, and TE. With the exception of KR domains, these domains have not been analyzed in previous studies of *Gambierdiscus* species; therefore, species comparisons are made only between the *G*. *polynesiensis* and *G*. *pacificus* transcriptomes from the current study.

#### Ketoreductase (KR)

The number of standalone KR domain proteins found in *G*. *polynesiensis* (11) and *G*. *pacificus* (12) was much smaller than that of standalone KSs presented above. This relative conservation of KR domains has been previously observed in dinoflagellates [29, 31, 33], including *Gambierdiscus* spp. [28]. All standalone KR domain proteins possessed the active site YxxxN present in PKSs, distinguishing them from classical short chain dehydrogenases (YxxxK). An exception is one sequence present in both *G*. *polynesiensis* and *G*. *pacificus* with a modified active site, LCAGN. Since the tyrosine (Y) is a critical residue for catalytic activity [64], the role of these sequences in PKS activity is uncertain. The PKS KR domains differed from the Type II fatty acid synthase KR (fabG) in these species (MUR4contig31717, Genbank Accession No. MT165603; TB92contig14168, Genbank Accession No. MT165604), which possessed the conserved active site YxxxK, specifically YGASK in both species. Both fabG sequences possessed a signal peptide consistent with plastid localization. All other standalone KR domains lacked signal peptides, transit peptides or peroxisome targeting signals when analyzed by signal or target. Deeploc assigned several to the peroxisome, but like in the KS domains, PTS motifs were absent, making the basis of the assignment uncertain.

Several multidomain sequences containing KR domains but lacking KS domains were found (Table 3), including an ER-KR sequence present in both species, ER-KR-PP-TE in *G*. *polynesiensis*, and AT-DH-KR[ER]KR-PP-TE in *G*. *pacificus*, which is 83% identical to *G*. *polynesiensis* contig3790 module 7, but missing the KS domain. Methyl transferase containing sequences with the structure MT-KR were present in both species, with similarity to lovastatin nonaketide synthase in *Symbiodinium microadriaticum* (Accession number OLP87649.1). *G*. *polynesiensis* expressed double (KR-KR) and triple KR sequences (KR-KR-KR) absent from *G*. *pacificus*.

#### Acyl Transferase (AT)

The number of *trans-*AT contigs far exceeded the number of *cis-*AT domains in both species: *G*. *polynesiensis* expressed 3 *cis-*AT and 28 *trans-*AT domains, while *G*. *pacificus* expressed 2 *cis-*AT and 27 *trans-*AT domains. *Cis-*AT domains in both species possessed the active site G**HS**xG, in which serine-histidine catalytic dyad is rigorously conserved, and a downstream conserved HAFH moiety is indicative of malonyl CoA specificity [65]. In both species, sequences with KS-AT-TE domain organization (TB92 contig55623 and MUR4 contig48074) possessed a modified downstream sequence of **K**AFH. This GHSLG…AFH sequence is conserved in burA-like sequences in other previously studied *Gambierdiscus* species and in *K*. *brevis*, and predicted to be specific for malonyl Co-A, as shown for the AT domain of the *Burkholderia* burA gene [66].

In contrast to *cis-*ATs, most *trans-*AT sequences contained the active site GLSLG, present in Type II FAS AT (fabD), and a downstream malonyl CoA-specific moiety of G(A/G)FH. Phylogenetic analysis of AT domains in *Symbiodinium* spp. showed similar distinctions in active sites between *cis-* and *trans-*AT sequences [31]. About half of the *trans-*AT contigs in both *Gambierdiscus* species included upstream ankyrin repeats that are likely involved in protein-protein interactions. These have been observed previously in *Gambierdiscus* spp. [28]. Both species also expressed sequences with multiple tandem AT domains: *G*. *polynesiensis* contig19885 (AT-AT-AT) and *G*. *pacificus* contig14691 (AT-AT), in which all domains possessed the *trans-*AT type active site G(L/F)SLG …(H/G)AFH. *Trans*-AT sequences lacked signal peptides, transit peptides or peroxisome targeting signals when analyzed by signalP, targetP, or Deeploc localization tools, and were assigned to cytoplasm by Deeploc. In contrast, AT domains for fatty acid biosynthesis, FabD (MUR4contig19339 and TB92contig7154), were identified as chloroplast-localized by targetP and Deeploc, and had a transit peptide cleavage site of V(V/T)L-AA with a FLFP motif 10 amino acids downstream of the cleavage site, similar to the FVAP motif described in peridinin dinoflagellates [58].

#### Dehydratase (DH)

DH are members of the hotdog fold superfamily of proteins. DH domains identified in modular PKSs were specific only to the level of hotdog fold superfamily, and not to the PKS DH (PF14765) in conserved domain searches. Ten modular DH domains were found among the PKSs in *G*. *polynesiensis* while only 3 standalone hotdog fold containing sequences were found. In *G*. *pacificus*, two modular PKS contained DH domains and 2 standalone hot dog sequences were expressed. The standalone sequences contained only partial hotdog fold domains, thus it is questionable if these are functional dehydratases. These sequences have little similarity with the type II fatty acid dehydratases in these species, FabZ (similarity <0.1), which unlike the partial hotdog folds have chloroplast transit peptides and are predicted to be plastid localized, consistent with other members of the Type II FAS.

#### Enoyl-ACP Reductase (ER)

ER domains of modular PKS and Type I FAS are members of the medium chain dehydrogenase reductase (MDR) protein family. In *G*. *polynesiensis*, 7 ER domains were found in modular PKSs (Table 3) while only 1 contig was identified as a standalone ER domain of a PKS (Cd08251; TB92contig63828). Several additional single domain sequences were identified as MDR family members, but their assignment to PKS is difficult, since the MDR family is large and involved in many cellular functions. The *G*. *pacificus* transcriptome had only 2 ERs in modular PKSs, while 2 standalone sequences were identified as MDR family members. It unclear from this analysis whether or not dinoflagellates possess standalone PKS ER domains (i.e., TB92contig63828 lacks the expected 5'-spliced leader or poly(A) tail to confirm it is not a partial assembly), but *trans-*ERs involved in PUFA and polyketide synthesis have been described in *Bacillus subtilis*, setting a precedence for *trans-*ER activity [67]. The standalone ER and single domain sequences identified as MDR differ significantly (similarity <10%) from the ER of Type II FAS (FabI) in both *G*. *polynesiensis* and *G*. *pacificus* transcriptomes (TB92contig8204 and MUR4contig49557), the latter of which are members of the short chain reductase family. Unlike candidate PKS ER sequences, FabI sequences possessed chloroplast transit peptides placing them in same compartment as other members of the Type II FAS complex.

Among the modular PKS ERs were two forms, canonical ER domains and ER domains embedded within a KR domain. The latter has been observed previously in *K*. *brevis* and *Ostreopsis*. In *G*. *polynesiensis*, the embedded ER form occurs only in the 7-module PKS (TB92contig3790). In *G*. *pacificus*, it is found only in MUR4contig9557, which has similarity to module 7 of the *G*. *polynesiensis* sequence. Alignment of modular ER domains with those from *K*. *brevis* reveals that the embedded ER domains are more similar between species than to the canonical ER domains in the same species. It is interesting to note that one of the *K*. *brevis* sequences containing the embedded ER is similar to *G*. *polynesiensis* modules 2-3-4 or 5-6-7 of the 7-module TB92contig3790 (described above in KS domain section). The canonical ER domains appear to be limited to NRPS/PKS sequences in both *Gambierdiscus* spp. and *K*. *brevis*.

#### Thioesterase domains (TE)

The *G*. *polynesiensis* transcriptome included 5 TE domains present in modular PKSs and 15 discrete TE domains. *G*. *pacificus* expressed 4 TEs in modular PKSs and 9 discrete TEs. TEs are members of the alpha-beta hydrolase-fold class of proteins, with a catalytic triad of Ser/Asp/His. *Cis-*acting TEs (TE I) serve to remove the growing chain from the PKS complex. Free standing, *trans-*acting TEs (TE II) associated with many PKS and NRPS play roles in editing and efficiency by cleaving incorrectly incorporated acyl groups from the ACP. Accordingly, TE I domains have deep substrate channels that accommodate the whole polyketide products, while a shallow cavity found in TE II proteins can accommodate only small acyl substrates, and substrate specificity may differ between individual proteins, e.g., for malonyl-ACP *vs* acetyl-ACP species [68]. Phylogenetic analysis placed most *Gambierdiscus* TE domains in a clade separate from bacterial TEII sequences (S2 Fig), with Gambierdiscus TEII (standalone) sequences generally falling in a clade separate from TEI (modular). All *Gambierdiscus* TEs possessed the conserved Gx**S**xG active site (present also in AT domains), and a conserved catalytic histidine. However, in some *Gambierdiscus* TEs the conserved histidine was not found within a conserved GxH motif previously identified in bacterial TEII and rat FAS TE, and sequences generally clustered accordingly. Many of the TE II standalone sequences occurred in pairs with high sequence similarity (>90%) in *G*. *polynesiensis* and *G*. *pacificus*, suggesting they represent homologs. No signal peptides, transit peptides or peroxisome targeting signals were found in standalone TEs, when analyzed by signalP, targetP, or Deeploc, respectively.

## Summary and conclusions

The identification of PKS genes in dinoflagellates has been hampered by their enormous genome sizes to obtain genome sequences, with the exception of *Symbiodinium* spp. [31, 69, 70, 71] and the parasite *Amoebophyya ceratii* [72], which have comparatively compact genome sizes. Most studies to date have therefore been limited to transcriptome analysis, which has yielded a consensus that dinoflagellates possess both Type I modular and standalone PKS domains that may function in concert with modular PKSs by providing activity *in trans*, and/or may form separate Type II -like complexes [73, 74]. In the current study we found that the highly ciguatoxic species, *G*. *polynesiensis*, both expressed a larger number of modular PKSs and those PKSs consisted of more modules than those in the non-ciguatoxic *G*. *pacificus*. The modular PKSs identified to date in any dinoflagellate are insufficient to account for the large and diverse polyketide compounds present in these species. The largest PKS identified in *Gambierdiscus* spp. is the 7-module (10K aa) PKS identified in two independent *G*. *polynesiensis* isolates and absent from other dinoflagellate species. *Symbiodinium minutum* clade B1 produces a similar sized, but unrelated, 8-module (10,601 aa) hybrid NRPS/PKS [30]. In both cases, the predicted products represent only a small portion of the carbon backbones of known metabolites present. How and if modular PKSs in dinoflagellates interact with specific *trans-*acting standalone domains remains unexplored. The expansion and diversification of standalone KS domains (close to 100 in all *Gambierdiscus* species) reveals the complexity that may be involved in assembling the dinoflagellate PKS machinery. Since the synthesis of well-studied polyketides in other eukaryotes involves PKSs in multiple cellular compartments, useful insight would come from knowing which modular and standalone domains occur in the same cellular compartment, enabling their *potential* interaction. To this end, in the current study we used several *in silico* tools to predict organellar location. After an enticing *in silico* lead suggested that most KS domains are localized to the peroxisome, careful analysis of known dinoflagellate peroxisome localized proteins and peroxisome targeting motifs led us to conclude that this prediction is unfounded, and the absence of organellar targeting signals suggests cytosolic localization of most of the standalone domains identified. Similarly, none of the modular PKS sequences appeared to be targeted to organelles. However, none of the modular PKS contigs possessed the 5’ dinoflagellate spliced leader to confirm that the complete 5’ end was assembled. Therefore, the absence of targeting signals may not accurately reflect the true disposition of the protein. Future efforts to characterize dinoflagellate PKS complexes will benefit from further insight into protein localization.

Although the 7-module PKS in *G*. *polynesiensis* was absent from other dinoflagellate species, we identified transcripts in several dinoflagellates that represent portions of the larger gene transcript. It is unclear from the current data if the absence of large PKSs is due to incomplete assemblies (inadequate sampling depth) or if the functions of the large PKS in *G*. *polynesiensis* are conducted in other species by cooperative action of smaller genes. The latter notion is supported by the *Symbiodinium* clade B1 genome, which encodes two adjacent genes on the same scaffold that together make up part of the 7-module PKS, with transcriptomic support. Not unlike *Gambierdiscus*, the comparative analysis of three *Symbiodinium* clades revealed that multiple gene duplication events, domain shuffling, and domain losses occurred even between closely related clades [31]. The application of new sequencing technologies that produce longer sequence reads and sufficient sequencing depth will be essential to confirm the PKS gene repertoire in dinoflagellates.

## Supporting information

S1 Fig Single domain KS homologs present in *G*. *polynesiensis* and *G*. *pacificus* identified by phylogenetic analysis. Single Domain KS Clade 1 (from Fig 1) illustrating pairs of highly similar sequences in *G*. *polynesiensis* and *G*. pacificus that appear to be homologs. Similar pairing of homologs is found in other clades.
(PPTX)

S2 Fig Maximum likelihood analysis of TE Domains in *G*. *polynesiensis* and *G*. *pacificus*. *Gambierdiscus* sequences generally clustered separately from bacterial TE II sequences. TE II (standalone) TEs cluster separately from TE I (modular) domains, an exception being two sequences with an internal TE domain with homology to burA (red).
(PPTX)

S1 Table Deeploc intracellular localization assignment of *G*. *polynesiensis* single domain KS. Assignment to peroxisome appears to be independent of clade. No evidence for C-terminal PTS1 signal (S/A/C-K/R/H-L/N).
(XLSX)

S2 Table Deeploc intracellular localization assignment of *G*. *pacificus* single domain KS. Assignment to peroxisome appears to be independent of clade. No evidence for C-terminal PTS1 signal (S/A/C-K/R/H-L/N).
(XLSX)

S3 Table Deeploc intracellular localization assignment of G. polynesiensis single domain KS. Assignment to peroxisome appears to be independent of clade. No evidence for C-terminal PTS1 signal (S/A/C-K/R/H-L/N).
(XLSX)

S4 Table Deeploc intracellular localization assignment of G. pacificus single domain KS. Assignment to peroxisome appears to be independent of clade. No evidence for C-terminal PTS1 signal (S/A/C-K/R/H-L/N).
(XLSX)

S5 Table Genbank accession numbers for peroxisomal proteins identified in G. polynesiensis TB92 and G. pacificus MUR4. (XLSX)
