# Supplementary figures and images for: Transcriptomic analysis of polyketide synthases in a highly ciguatoxic dinoflagellate, Gambierdiscus polynesiensis and low toxicity Gambierdiscus pacificus, from French Polynesia
Source: PLoS One. 2020 Apr 15;15(4):e0231400. doi: 10.1371/journal.pone.0231400 (PMC7159223; doi:10.1371/journal.pone.0231400)

## Slide 1
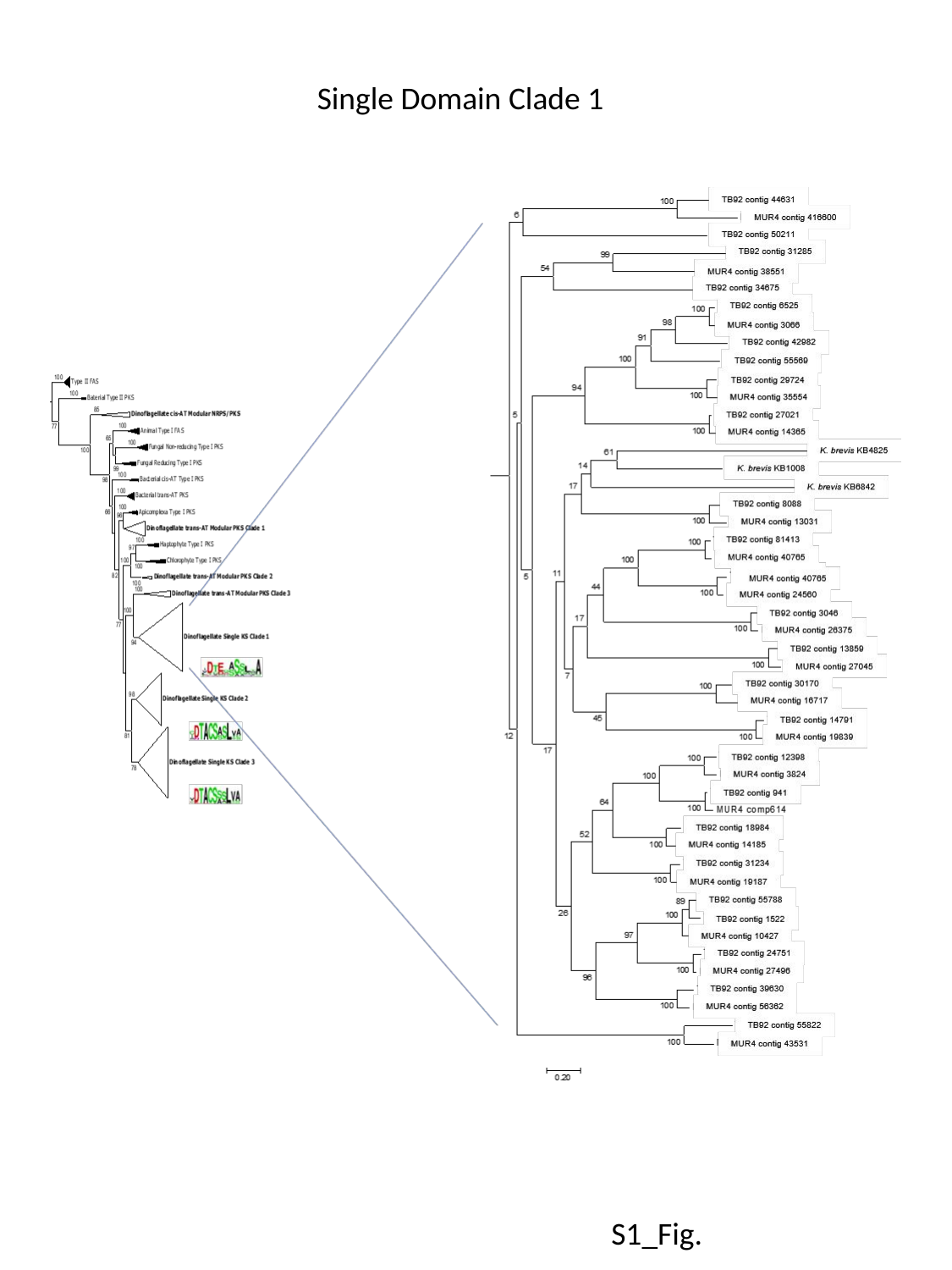

Single Domain Clade 1
S1_Fig.

## Slide 2
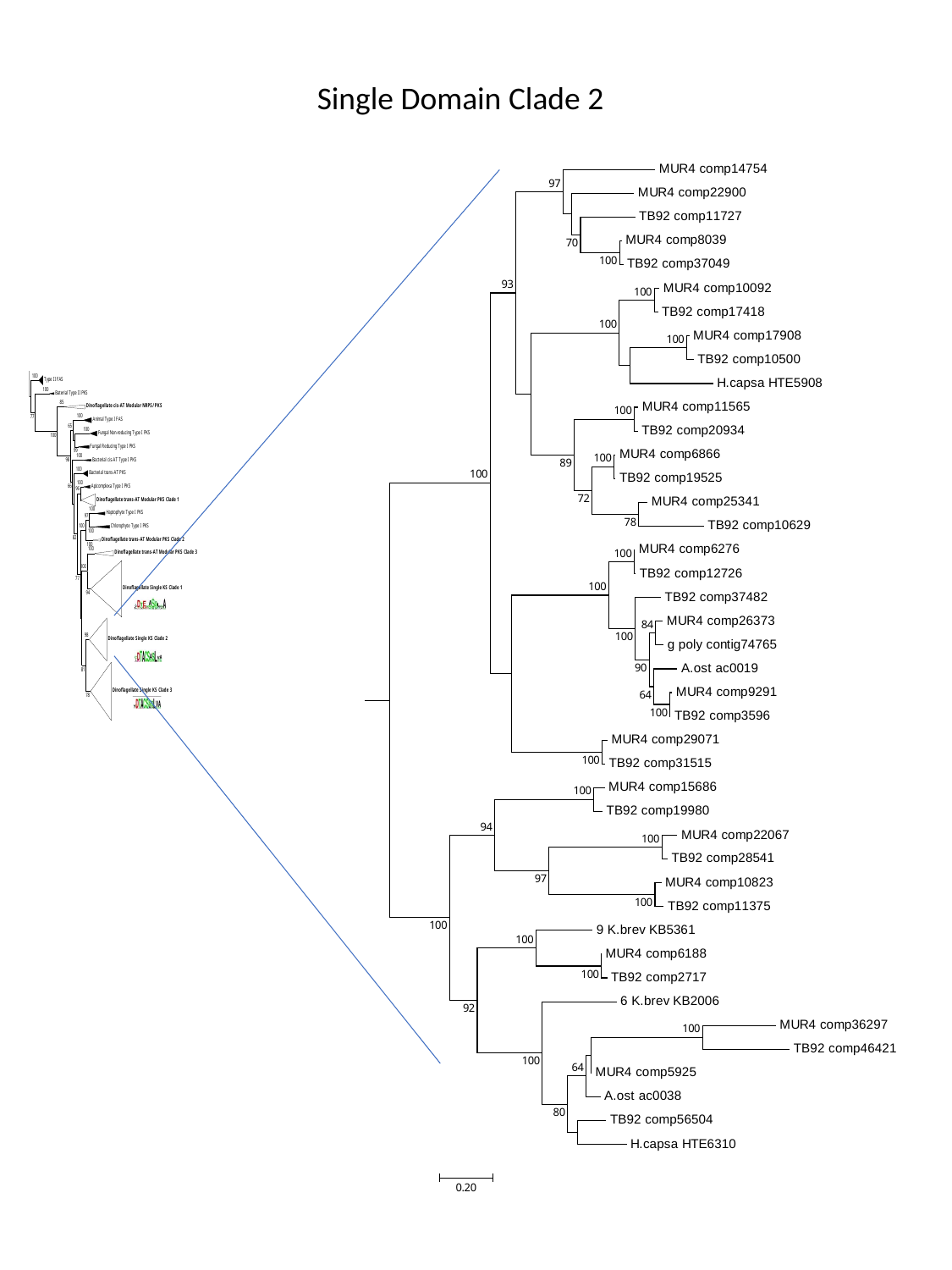

Single Domain Clade 2

## Slide 3
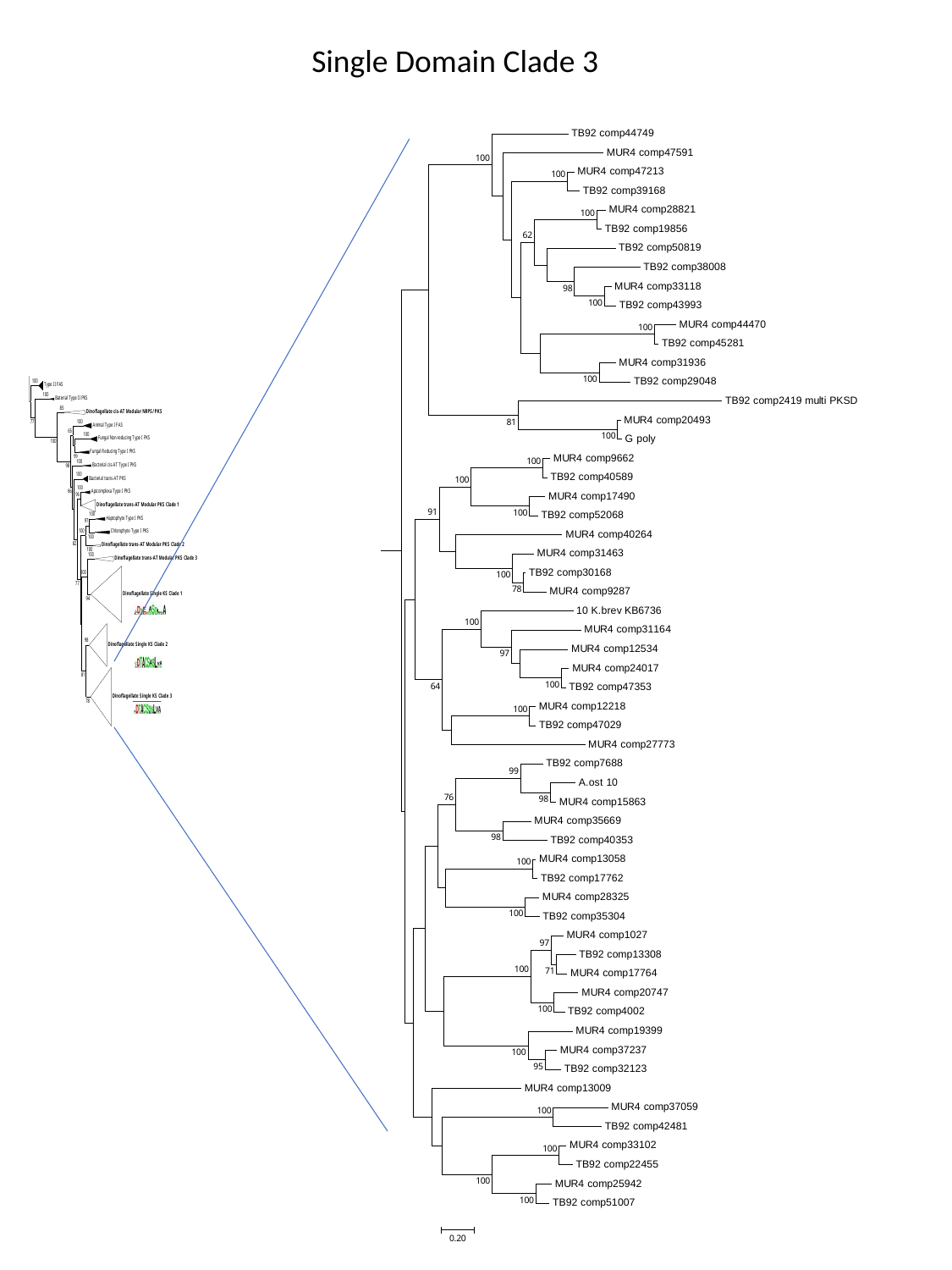

Single Domain Clade 3

Supplement: S1 Fig — Single Domain KS Clade 1 (from Fig 1) illustrating pairs of highly similar sequences in G. polynesiensis and G. pacificus that appear to be homologs. Similar pairing of homologs is found in other clades. (PPTX) [file pone.0231400.s001.pptx]
