# Supplementary material for: Transcriptomic analysis of polyketide synthases in a highly ciguatoxic dinoflagellate, Gambierdiscus polynesiensis and low toxicity Gambierdiscus pacificus, from French Polynesia
Source: PLoS One. 2020 Apr 15;15(4):e0231400. doi: 10.1371/journal.pone.0231400 (PMC7159223; doi:10.1371/journal.pone.0231400)
Supplement: S2 Fig — Gambierdiscus sequences generally clustered separately from bacterial TE II sequences. TE II (standalone) TEs cluster separately from TE I (modular) domains, an exception being two sequences with an internal TE domain with homology to burA (red). (PPTX) [file pone.0231400.s002.pptx]

## Slide 1
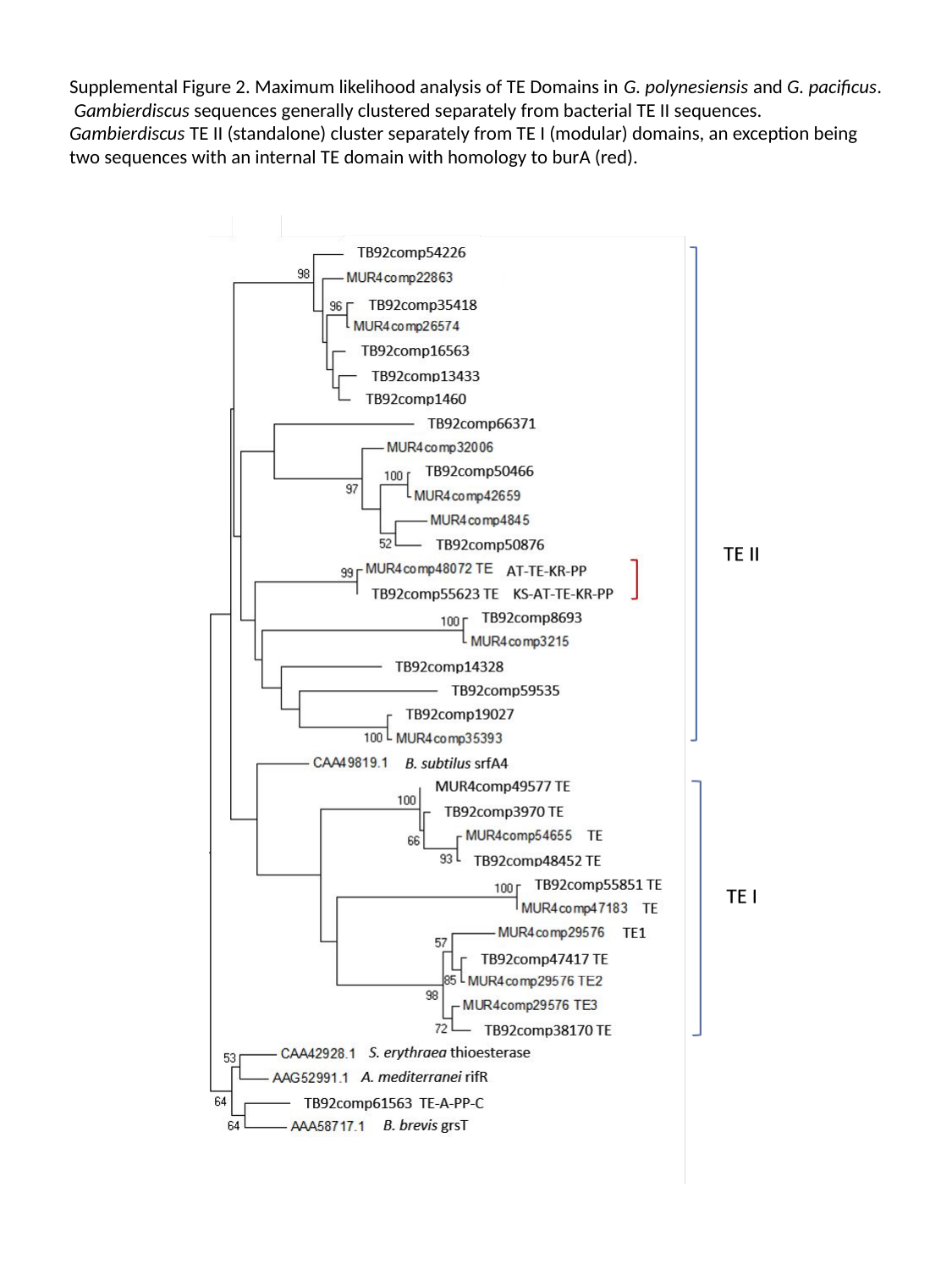

Supplemental Figure 2. Maximum likelihood analysis of TE Domains in G. polynesiensis and G. pacificus. Gambierdiscus sequences generally clustered separately from bacterial TE II sequences. Gambierdiscus TE II (standalone) cluster separately from TE I (modular) domains, an exception being two sequences with an internal TE domain with homology to burA (red).
